# Perceived Social Integration Predicts Engagement and Responsiveness to Positive Events: Test of Age Moderation

**DOI:** 10.1093/geroni/igaa057.1278

**Published:** 2020-12-16

**Authors:** Patrick Klaiber, David Almeida, Nancy Sin

**Affiliations:** 1 University of British Columbia, Vancouver, British Columbia, Canada; 2 Pennsylvania State University, University Park, Pennsylvania, United States

## Abstract

Social Integration has important implications for health and well-being during adulthood. Being socially integrated might be an important resource that helps people to regularly engage in daily positive events. With older age, this resource might become increasingly important. However, being well socially integrated might also mean that people are certain that they experience more positive events in the future and thus, respond with less positive affect to any given positive event. We examined perceived social integration as a predictor of engagement and responsiveness to positive events using data from the National Study of Daily Experiences 2. 1904 adults (Mean age = 56.25, min = 33, max = 84) reported their daily positive affect and daily positive events during 8 consecutive days of telephone interviews. Perceived social integration was assessed at baseline. Adults higher in social integration experienced daily positive events more frequently (b = 0.01, SE = 0.003, p < .001), but showed less of an increase in positive affect on days with more-than-usual positive events (b = -0.003, SE = 0.001, p = .030). These models controlled for the Big Five personality traits, purpose in life, demographic variables and same-day occurrence of stressors. Age did not moderate the present associations. The present findings imply that social integration might be an important contributor to experiencing more positive events across adulthood. Being better socially integrated might also lead to responding with less positive emotions to any given positive event.

